# The effects of changes in distance to nearest health facility on under-5 mortality and health care utilization in rural Malawi, 1980–1998

**DOI:** 10.1186/s12913-020-05738-w

**Published:** 2020-09-24

**Authors:** John P. Quattrochi, Kenneth Hill, Joshua A. Salomon, Marcia C. Castro

**Affiliations:** 1 Department of Public Health, Simmons University, 300 The Fenway, Boston, MA 02115 USA; 2 Center for Health Policy and Center for Primary Care and Outcomes Research, Stanford University, 615 Crothers Way, Stanford, CA 94305 USA; 3 Department of Global Health and Population, Harvard T.H. Chan School of Public Health, 655 Huntington Ave, Boston, MA 02115 USA

**Keywords:** Under-5 mortality, Utilization, Maternal health, Distance, Service availability, Malawi

## Abstract

**Background:**

Despite important progress, the burden of under-5 mortality remains unacceptably high, with an estimated 5.3 million deaths in 2018. Lack of access to health care is a major risk factor for under-5 mortality, and distance to health care facilities has been shown to be associated with less access to care in multiple contexts, but few such studies have used a counterfactual approach to produce causal estimates.

**Methods:**

We combined retrospective reports on 18,714 births between 1980 and 1998 from the 2000 Malawi Demographic and Health Survey with a 1998 health facility census that includes the date of construction for each facility, including 335 maternity or maternity/dispensary facilities built in rural areas between 1980 and 1998. We estimated associations between distance to nearest health facility and (i) under-5 mortality, using Cox proportional hazards models, and (ii) maternal health care utilization (antenatal visits prior to delivery, place of delivery, receiving skilled assistance during delivery, and receiving a check-up following delivery), using linear probability models. We also estimated the causal effect of reducing the distance to nearest facility on those outcomes, using a two-way fixed effects approach.

**Findings:**

We found that greater distance was associated with higher mortality (hazard ratio 1.007 for one additional kilometer [95%CI 1.001 to 1.014]) and lower health care utilization (for one additional kilometer: 1.2 percentage point (pp) increase in homebirth [95%CI 0.8 to 1.5]; 0.8 pp. decrease in at least three antenatal visits [95% CI − 1.4 to − 0.2]; 1.2 pp. decrease in skilled assistance during delivery [95%CI − 1.6 to − 0.8]). However, we found no effects of a decrease in distance to the nearest health facility on the hazard of death before age 5 years, nor on antenatal visits prior to delivery, place of delivery, or receiving skilled assistance during delivery. We also found that reductions in distance decrease the probability that a woman receives a check-up following delivery (2.4 pp. decrease for a 1 km decrease [95%CI 0.004 to 0.044]).

**Conclusion:**

Reducing under-5 mortality and increasing utilization of care in rural Malawi and similar settings may require more than the construction of new health infrastructure. Importantly, the effects estimated here likely depend on the quality of health care, the availability of transportation, the demand for health services, and the underlying causes of mortality, among other factors.

## Background

Despite important progress, the burden of under-5 mortality remains unacceptably high, with an estimated 5.3 million deaths in 2018 [1]. The Sustainable Development Goals include ending preventable deaths of children under 5 by 2030 [2]. Lack of access to health care is a major risk factor for under-5 mortality, and distance to health care facilities has been shown to be associated with less access to care in multiple contexts [3–6]. Pregnant women living farthest from heath facilities are generally the least likely to give birth in a facility, and least likely to utilize antenatal care [3, 5–7]. Their children tend to be at higher mortality risk than those of mothers living closer to facilities. There remains a question, however, about the extent to which this relationship is causal. Several studies have found no relationship between distance to nearest facility and health outcomes or utilization [8–10]. In some contexts, other barriers to access, such as financial cost, social support, time availability, and caregiver autonomy, may be more important [11].

Malawi has reduced its under-5 mortality rate from 250 per 1000 live births in 1990 to 54 in 2018, a 78% reduction, despite an HIV/AIDS epidemic that peaked at approximately 15% prevalence [12]. While health policy prior to Malawi’s first democratic elections in 1994 tended to favor private provision of health care, over the last 24 years Malawian policymakers have put forward bold programs to make health care more accessible, including 1994’s National Safe Motherhood Program and 2012’s Presidential Initiative for Safe Motherhood [13–19].

In this paper, we estimated the associations between distance to nearest health facility and (i) under-5 mortality, and (ii) maternal health care utilization (place of delivery, receiving a check-up following delivery, number of antenatal visits prior to delivery, or receiving skilled assistance during delivery). We also took a two-way fixed effects approach to estimate the causal effect of reductions in distance from the construction of new facilities on those outcomes.

## Methods

### Data

#### Health facility data

A national census of health facilities in 1998 carried out by the Malawi Ministry of Health and the Japanese International Cooperation Agency (MOH & JICA 1999) listed 719 facilities in operation. For most facilities, the data included the date of construction (589 out of the 719 (82%)), and for all facilities it included the GPS coordinates, the facility type, principle funder, and owner.

**Fig. 1:**
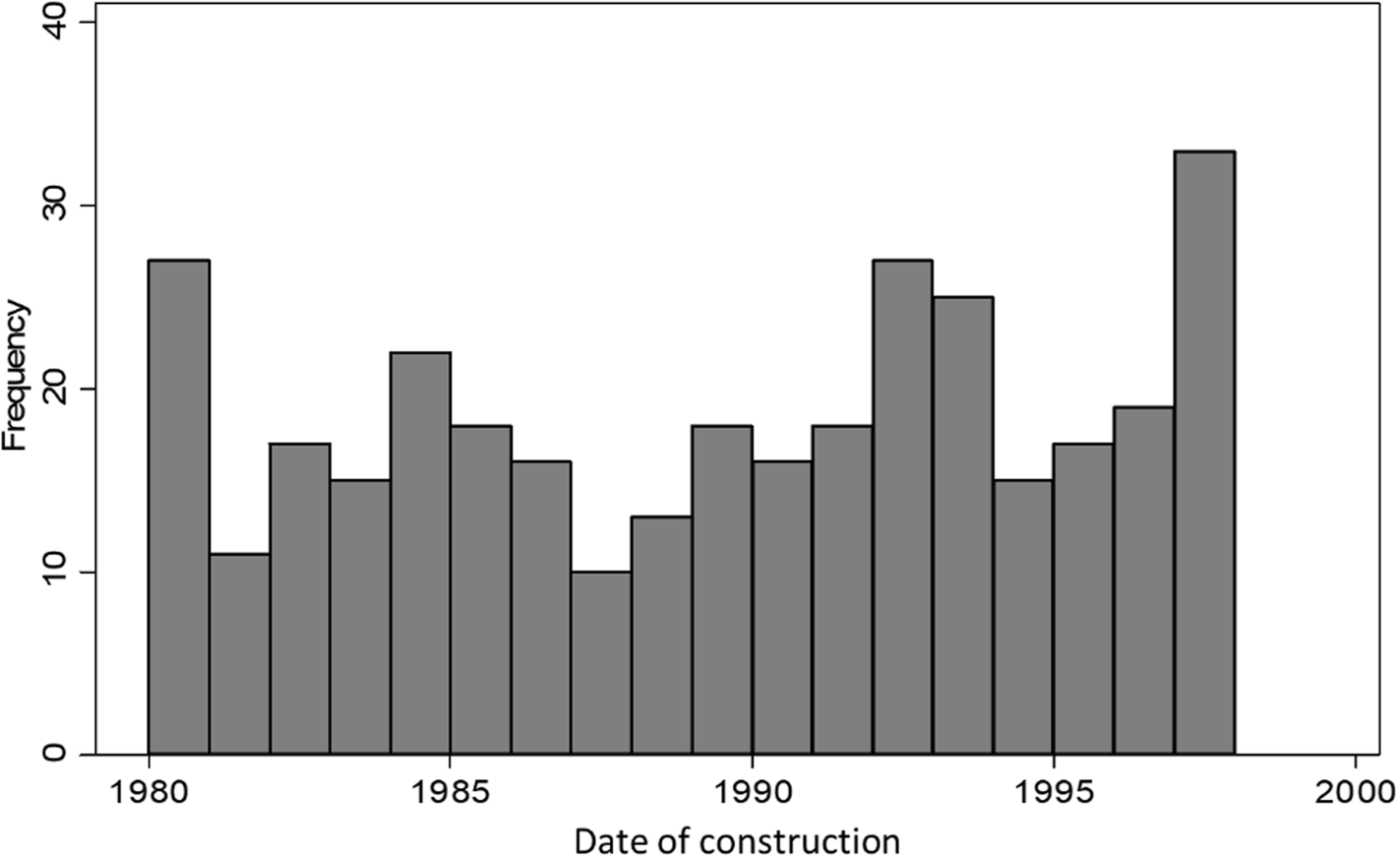
Number of new health facilities constructed per year in Malawi, 1980 to 1998. Notes: data from national census of health facilities in 1998 carried out by the Malawi Ministry of Health and the Japanese International Cooperation Agency

The dates of construction ranged from 1889 (when the first missionary hospital was built) to 1998. We restricted our analysis to the period from 1980 to 1998 to reduce recall bias in the mortality data (described below). Of the 589 facilities for which we have date of construction, 337 (57%) were built between 1980 and 1998. There was no apparent temporal trend in the number of facilities built per year; the number ranged from a minimum of 10 in 1987 to a maximum of 32 in 1998 (Fig. 1).

Of the 337 new facilities, 92% were one of two types: 135 (40%) are classified as “dispensary” and 175 (52%) are classified as “dispensary/maternity.” We restricted our analysis to these facilities. Dispensaries are permanent structures from which drugs are distributed. They provide outpatient care and may contain holding beds. Dispensary/maternities are similar but provide more extensive services to expectant mothers (antenatal, delivery, and postnatal care). The other facility types are district hospital, hospital, mental hospital, primary health center and urban health center. These are almost exclusively located in urban areas.

We assumed that those facilities missing the date of construction (130 out of 719), were built prior to 1980. If some of these facilities were in fact built between 1980 and 1998, then some births will be erroneously coded as being closer to a health facility than they actually were. This will bias the effect estimate towards the null if the true effect of being closer to a health facility is to reduce mortality or increase utilization.

#### Mortality and utilization data

**Fig. 2:**
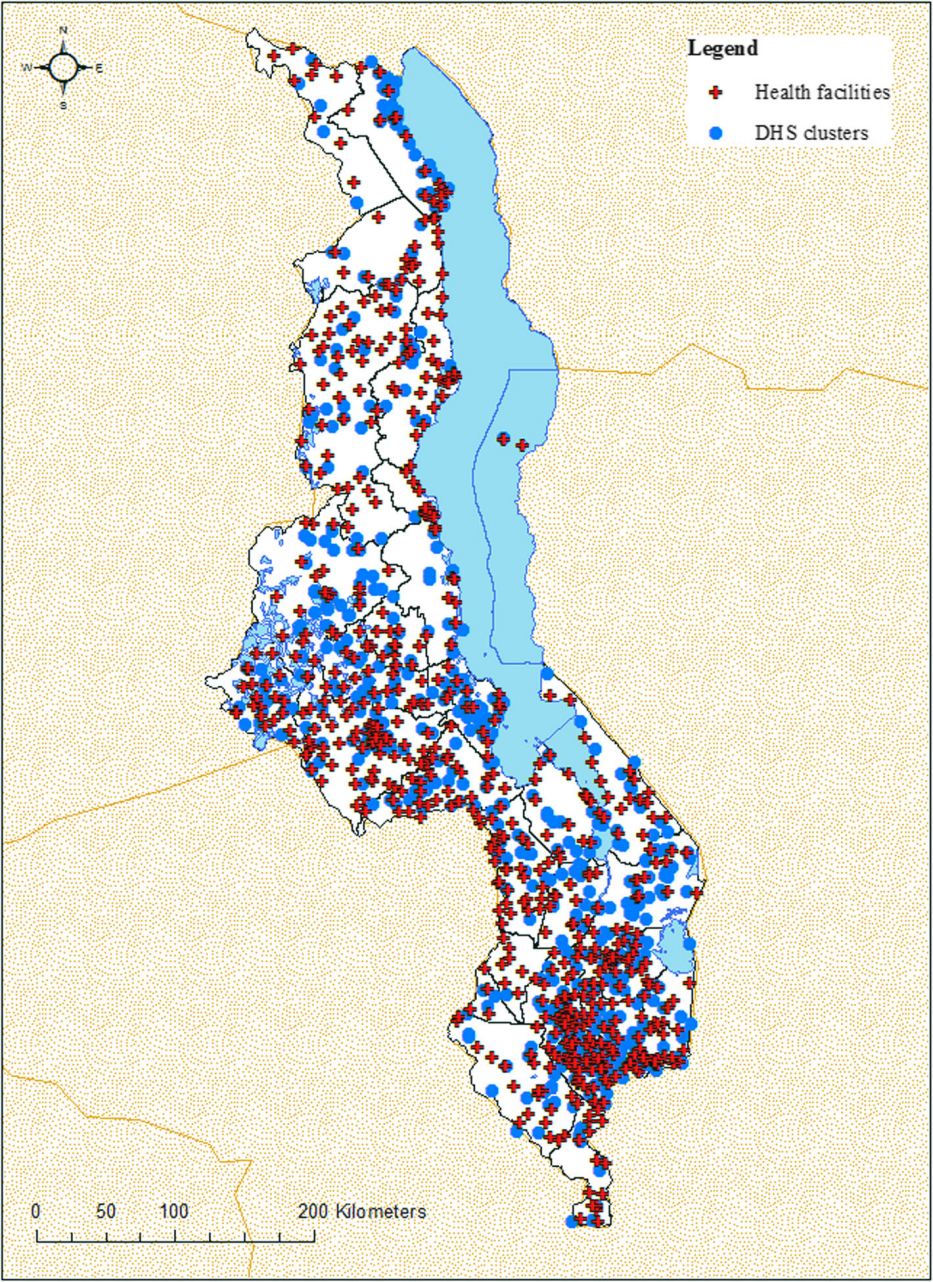
Map of health facilities and enumeration areas in Malawi. Notes: map created by the authors using data from 1998 Malawi Health Facility Census and the 2000 Malawi Demographic and Health Survey

The data on under-5 mortality and health care utilization came from the 2000 Malawi Demographic and Health Survey (MDHS), a nationally representative survey targeting all resident women aged 15–49 [20]. Variables collected include the date of birth and date of death (if applicable) for all children ever born to respondents (*n* = 40,221 children). The data also included GPS coordinates for centroids of MDHS enumeration areas, which we refer to as villages in the remainder of the paper (Fig. 2). Enumeration areas were based on the 1998 census, which identified 9213 in total. Rural enumerations areas have populations of between 800 and 1200 persons.

For births in the 5 years prior to the survey, the MDHS also contains information on the following utilization outcomes: (1) place of delivery, (2) receiving a check-up following delivery, (3) number of antenatal visits prior to delivery, and (4) receiving skilled assistance during delivery.

Migration has the potential to cause measurement error in the treatment variable since mothers’ residences in 2000 may not be in the same location as their residences in previous years. We restricted our analysis to rural births that occurred at the same location where the mother was living at the time of the survey, as reported in an MDHS question about length of time at one’s current residence.

### Operationalization of treatments and outcomes

The primary outcome of interest was the hazard of death between birth and age five, estimated from retrospective birth histories in the MDHS that included the date of birth and date of death for each child. Children still alive and under age five at the time of the survey were right-censored (i.e. they have missing survival data between the age at which the survey occurred and age five). Additional file 1 includes tests showing that recall bias is not a concern in these data.

For the causal analysis, the treatment of interest is the reduction in distance to the nearest health facility caused by the construction of a new facility, conditional on distance to the nearest health facility prior to the construction of a new facility. In addition to models in which the linear reduction in distance is the treatment variable, in other models we use multiple treatment variables to reflect the intuition that the benefit of a new facility depends on both the distance from the village to the old facility and the distance from the village to the new facility.

For those models, we created four distance categories (< 2 km, 2-5 km, 5-10 km, and > 10 km to nearest facility), which correspond to six possible treatments, each representing a move from one distance category to a nearer distance category: (1) > 10 km to 5-10 km, (2) > 10 km to 2-5 km, (3) > 10 km to < 2 km, (4) 5-10 km to 2-5 km, (5) 5-10 km to < 2 km, and (6) 2-5 km to < 2 km. The reference category is no change in distance category.

For each village we calculated the distance to the nearest health facility in each year from 1979 to 1998. When a new facility was built, resulting in a change in distance to nearest facility, we assigned that village to one of the above distance change categories for all remaining years. We linked the change in distance category (including no change) for each village-year to each child-year in the mortality dataset. In villages where no new facility was built, all person-time is assigned to the ‘no change’ category. In villages where a new facility is built, all person-time before the facility is built is assigned to the ‘no change’ category. All person-time after the facility is built is assigned to the appropriate distance change category (e.g. > 10 km to 5-10 km). If a facility is built during a child’s life from age 0 to 5, the portion before the facility was built was assigned to the ‘no change’ category, and the portion after the facilty was built was assigned to the appropriate distance change category. We linked the change in distance category (including no change) for each village-year to each child-year in the mortality dataset. The dates of construction did not include the month of construction. Therefore, to avoid over-estimating exposure to new facilities, construction was assumed to have occurred on December 31.

For the secondary outcomes, we used the linear reduction in distance as our treatment variable. We do not use the multiple category distance reduction variable due to much smaller sample sizes.

### Identification strategy and statistical analysis

An ideal study of the effects of new health facilities on under-5 mortality would randomly assign villages to receive a new health facility. By comparing the mortality rates before and after health facility construction in villages that did receive a facility to those that did not, the average treatment effect of a new facility could be easily calculated. In the current study, the location for new facilities may be endogenous to the under-5 mortality rate. If one were to simply carry out a cross-sectional comparison of areas with new health facilities to those without, it is unclear which direction the bias would take. For example, if facilities tend to be located in areas with higher disease burden, they may be positively associated with mortality, even if they have a beneficial effect. Conversely, if they tend to be built in wealthier areas, they may appear to be negatively associated with mortality, even if they have no effect.

We estimated the association between distance to nearest facility and mortality or utilization as a first step to investigating causality. Mortality was measured as survival time at the child-level, and many observations were right-censored (i.e. children were under five and still alive at the time of the survey). Therefore, we fit semi-parametric Cox proportional hazards models [21, 22]. Models 1–4 used linear distance to test for a non-linear relationship between distance and mortality. These include models with and without dummies for year (*n* = 18; to capture temporal trends in mortality unrelated to new facilities) and month (*n* = 11; to capture seasonality in mortality). Model 4 adds controls for child and mother characteristics. As a sensitivity analysis, we run the same models with the logarithm of distance rather than linear distance (see Additional file 1). For our secondary outcomes, which are binary and thus not right-censored, we used linear probability models.

To estimate the causal effect of changes in distance on mortality, we used stratified Cox models, with each stratum corresponding to one village. This controls for time-invariant characteristics of each village, and uses *within* village variation in distance and mortality to estimate effects. In some models, we again added year dummies to capture temporal trends; this is sometimes referred to as a two-way fixed effects model (two-way referring to time and space) [23]. We thus estimated the multiplicative change in the hazard ratio for mortality within these villages, before and after changes in distance. For our secondary outcomes, we take a similar approach, but using linear probability models rather than Cox models. We included fixed effects for each village and each year.

One key assumption, inherent in Cox models, is that the hazards are proportional. We tested this assumption in two ways (Additional file 1). First, we tested for non-zero slope in a generalized linear regression of the scaled Schoenfeld residuals on time [24]. Second, because that test can be “over-powered” – with many observations it may classify substantially insignificant changes in the hazard ratio as statistically significant -- we visually assessed plots of the scaled Schoenfeld residuals for the covariates that the test identified as violating the proportional hazards assumption [25].

*P*-values for distance reduction coefficients in the categorical distance models were adjusted for multiple testing using a false discovery rate procedure [26].

Ethical approval was obtained from Simmons University Institutional Review Board.

## Results

There were a total of 40,306 births reported in the 2000 MDHS. Of those births, 18,714 were eligible for the mortality analysis, meaning that they occurred in a rural area between 1980 and 1998 to a woman in a village who reported living in the same village since at least the date of birth (Table 1). Of the mothers in the mortality analysis sample, 43.8% had no education, 55.0% had primary education, and 1.2% had secondary education or higher. For the utilization analysis, the number of eligible births ranged from 2333 to 4926.

**Table 1:** Descriptive statistics

	Obs.	*n* or mean	% or SD	Min	Max
Total births	40,306				
Rural births	33,477				
Eligible rural births	18,714				
*Primary outcomes, exposure, and control variables among eligible rural births*					
Child deaths	18,714	3887	20.8%		
Distance to nearest health facility at birth (meters)	18,714	6182	4744	305	47,442
Births to women with no education	18,714	8197	43.8%		
Births to women with primary education	18,714	10,293	55.0%		
Births to women with secondary education or higher	18,714	225	1.2%		
First birth	18,714	3743	20.0%		
Twin	18,714	749	4.0%		
Births to a mother under 19 years old	18,714	3668	19.6%		
Births to a mother over 35 years old	18,714	2133	11.4%		
*Secondary outcomes (only asked to mothers of children under 5 years old)*					
Home birth	4889	2302	47.1%		
At least three antenatal visits prior to delivery	2333	1316	56.4%		
Skilled assistance during delivery	4926	2565	52.1%		
Check-up following delivery	2333	167	7.2%		

Notes: data from 2000 Malawi Demographic and Health Survey. Eligible rural births are those that occurred between 1980 and 1998 to a woman who reports having lived in the same village when she gave birth as she did at the time of the interview

**Fig. 3:**
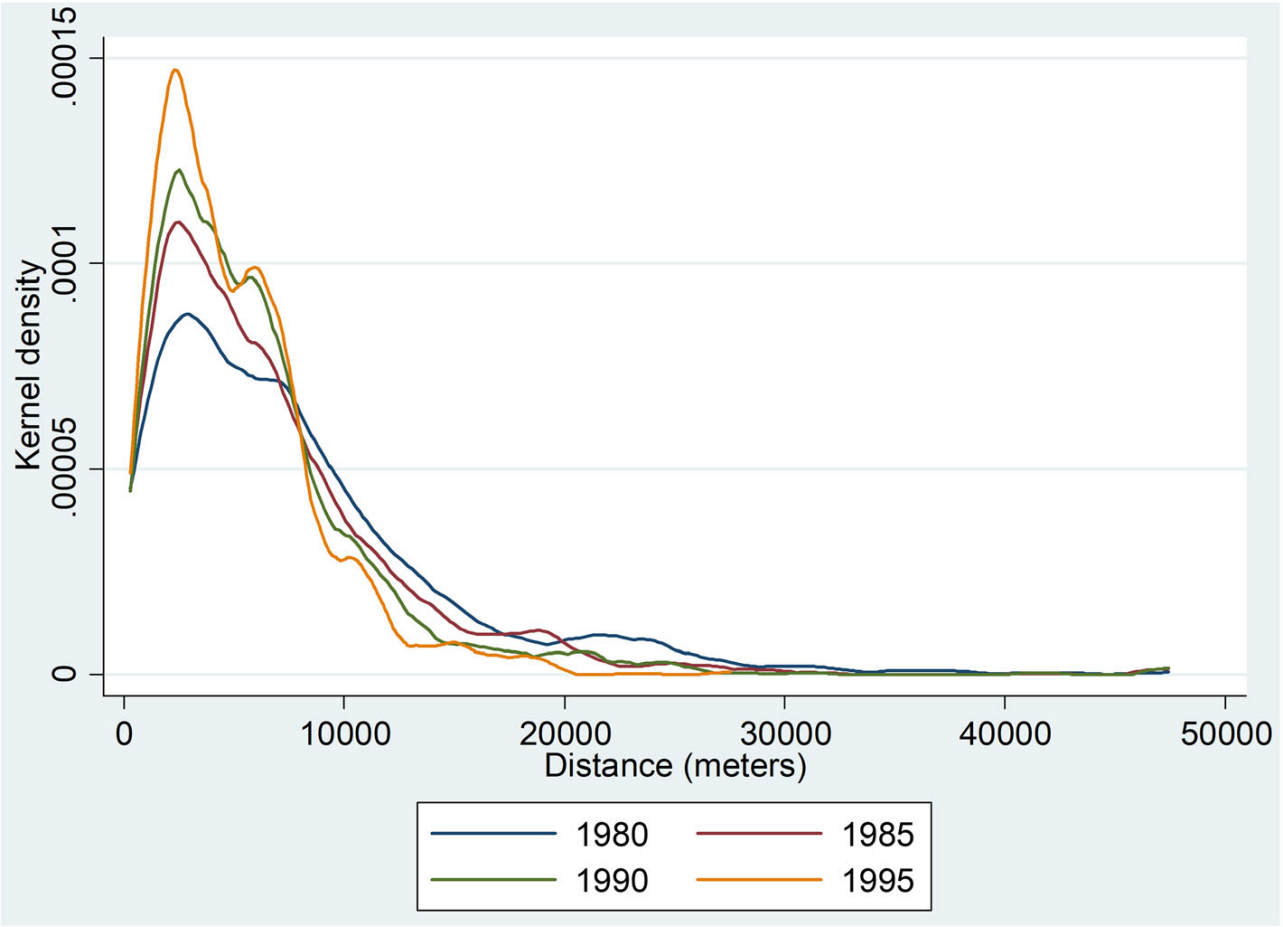
Distribution of births by distance from village to nearest health facility, selected years of birth. Notes: Kernel densities of birth in given year by distance from village to nearest health facility. Data from 2000 Malawi Demographic and Health Survey and 1998 Malawi Health Facility Census

**Fig. 4:**
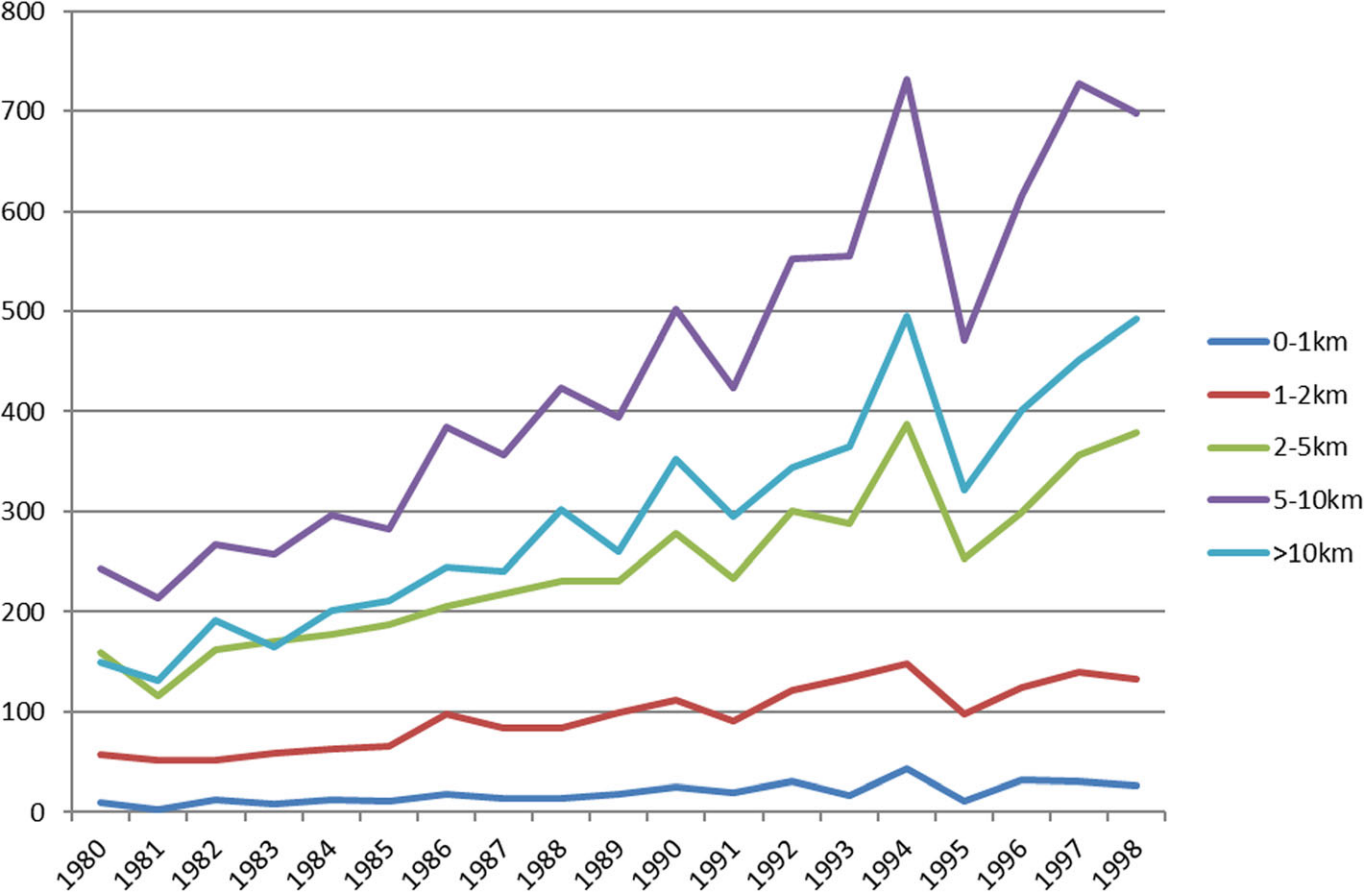
Births per year by distance to nearest health facility in analysis sample. Notes: Data from 2000 Malawi Demographic and Health Survey (DHS) and 1998 Malawi Health Facility Census. Age-heaping in 1994 likely due to the additional DHS questions required of children under 5 years old

Kernel density plots of births by distance to nearest facility for selected years show that the average distance has been decreasing over time, driven in particular by facilities 10-20 km from a village being replaced by facilities less than 10 km away from a village (Fig. 3). Overall, the modal distance is 5-10 km, and very few births occurred within 1 km of a health facility (Fig. 4). Turning to the number of births per year, the number was higher in more recent years because the population was growing rapidly and the data come from women who were aged 15–49 in 2000. The sharp decrease in births in 1995 is almost certainly due to misreporting of 4 year olds as 5 year olds. This means that for a small number of child-years, children will be coded as being untreated when they were in fact treated. If the true effect of health facilities is protective, then this will bias estimates toward the null.

Each village contributed an average of 1643 child-months (137 child-years) to the analysis (min 32; max 4493) (Table 2). On average, 1289 of those child-months (78%) occurred prior to the construction of a new facility. Of the child-months that occurred after the construction of a new facility, relatively few were contributed by villages in which the distance to nearest facility decreased from 2 to 5 km to less than 2 km (only six villages fell into this category). Thus the statistical analysis that follows may have relatively little power to detect effects from changes to less than 2 km.

**Table 2:** Person-time contributed per village, by treatment category

	No. villages	Mean	SD	Min.	Max.
Total child-months	449	1643	797	32	4493
Child-months prior to new facility	449	1289	893	1	4106
*Original treatments*					
Child-months after > 10 km to 5-10 km	57	949	737	2	2884
Child-months after > 10 km to 2-5 km	24	1564	1128	102	4380
Child-months after > 10 km to < 2 km	9	1327	769	1	2273
Child-months after 5-10 km to 2-5 km	41	957	625	1	2441
Child-months after 5-10 km to < 2 km	14	815	470	91	1760
Child-months after 2-5 km to < 2 km	6	783	547	306	1715
Year of first birth in village	449	1981	2.3	1980	1997
Year of last birth in village	449	1998	0.23	1996	1998

Notes: “village” refers to rural enumeration areas in the 2000 Malawi Demographic and Health Survey. Child-months are from children born between 1980 and 1998 to mothers who, at the time of interview, continued to reside in the village where the child was born

In all four models testing an association between distance and mortality, we found a significant relationship at *p* < 0.05. (Table 3) The bivariate model with linear distance showed that each additional kilometer was associated with an 1.1% increase in the hazard of death (95%CI 0.5 to 1.8%). Adding controls for year and month reduced the hazard ratio slightly to 1.007 (95%CI 1.001 to 1.014). Similar results were found after adding controls for child and mother characteristics (column 4) and using log (distance) instead of linear distance (see Additional file 1).

**Table 3:** Association between distance to nearest facility and under-5 mortality

Variables	(1) U5M	(2) U5M	(3) U5M	(4) U5M
Distance (km)	1.011*** (1.005–1.018)	1.007** (1.001–1.014)	1.007** (1.001–1.014)	1.007** (1.000–1.013)
First birth				1.000 (0.909–1.100)
Twin				2.898*** (2.579–3.256)
Mother under 19				1.436*** (1.310–1.573)
Mother over 35				1.025 (0.920–1.143)
Mother has primary education				0.813*** (0.763–0.866)
Mother has secondary education				0.381*** (0.242–0.599)
Year dummies?	NO	YES	YES	YES
Month dummies?	NO	NO	YES	YES
Total children	18,714	18,714	18,714	18,714
Total deaths	3887	3887	3887	3887
Total child-months	737,547	737,547	737,547	737,547

*Notes*: *U5M* under-5 mortality

Hazard ratios (95% confidence intervals) from proportional hazards models. Distance is linear distance to nearest health facility from village centroid. The coefficient on the distance variable represents the HR for a one-kilometer increase in distance. The reference category for mother’s education is ‘less than primary’, and for mother’s age is ‘19–35 years old’

*** *p* < 0.01

** *p* < 0.05

* *p* < 0.1

We found no statistically significant effect of reductions in linear distance to nearest health facility on under-5 mortality, using Cox models stratified by village (Table 4). The effect was marginally significant (*p* < 0.10) when linear distance was the only variable, but no longer significant after we added controls for year, month, and mother and child characteristics.

**Table 4:** The effect of changes in distance to nearest health facility on under-5 mortality, linear distance

Variables	(1) U5M	(2) U5M	(3) U5M	(4) U5M
Distance (km)	1.013* (1.000–1.025)	0.994 (0.981–1.007)	0.994 (0.981–1.007)	0.994 (0.981–1.007)
First birth				1.011 (0.918–1.113)
Twin				3.038*** (2.680–3.443)
Mother under 19				1.389*** (1.263–1.526)
Mother over 35				0.995 (0.888–1.113)
Mother has primary education				0.865*** (0.804–0.931)
Mother has secondary education				0.453*** (0.284–0.722)
Year dummies?	NO	YES	YES	YES
Month dummies?	NO	NO	YES	YES
Total children	18,714	18,714	18,714	18,714
Total deaths	3887	3887	3887	3887
Total child-months	737,547	737,547	737,547	737,547

*p < 0.1

***p < 0.01

Notes: Hazard ratios (HR) from Cox proportional hazards models, with baseline hazard stratified by village of birth (*n* = 449) The coefficient on the distance variable represents the HR for a one-kilometer increase in distance. The reference category for mother’s education is ‘less than primary’, and for mother’s age is ‘19–35 years old’

Using categorical variables to account for differences in initial distance to nearest health facility in Cox models stratified by village, we again found no statistically significant effect of reductions in distance to nearest health facility on under-5 mortality (Table 5). Reductions in distance from 5 to 10 km to 2-5 km caused a 37.5% reduction in the hazard of mortality in the model with no controls for year, seasonality, or mother and child characteristics; however, once those controls were added, there was no effect from any category of distance reduction.

**Table 5:** The effect of changes in distance to nearest health facility on under-5 mortality, categorical distance

Variables	(1) U5M	adj. *p*-value	(2) U5M	adj. *p*-value	(3) U5M	adj. *p*-value
> 10 km to 5-10 km	0.861 (0.671–1.106)	0.49	1.072 (0.828–1.387)	0.82	1.084 (0.837–1.405)	0.70
> 10 km to 2-5 km	1.061 (0.727–1.550)	0.91	1.314 (0.892–1.936)	0.50	1.354 (0.914–2.007)	0.39
> 10 km to < 2 km	0.818 (0.412–1.626)	0.85	1.007 (0.502–2.017)	0.99	0.922 (0.461–1.846)	0.82
5-10 km to 2-5 km	0.625*** (0.485–0.806)	0.002	0.809 (0.621–1.054)	0.50	0.789 (0.604–1.029)	0.39
5-10 km to < 2 km	0.701 (0.466–1.054)	0.26	0.917 (0.605–1.390)	0.82	0.891 (0.588–1.351)	0.70
2-5 km to < 2 km	0.981 (0.485–1.984)	0.96	1.253 (0.615–2.555)	0.82	1.336 (0.660–2.705)	0.70
First birth					1.011 (0.918–1.114)	
Twin					3.050*** (2.691–3.457)	
Mother under 19					1.389*** (1.264–1.527)	
Mother over 35					0.995 (0.889–1.114)	
Mother primary ed.					0.865*** (0.804–0.931)	
Mother secondary ed.					0.452*** (0.283–0.720)	
Year dummies?	NO		YES		YES	
Month dummies?	NO		YES		YES	
Total children	18,714		18,714		18,714	
Total deaths	3887		3887		3887	
Total child-months	737,547		737,547		737,547	

***p < 0.01

Notes: Hazard ratios from Cox proportional hazards models, with baseline hazard stratified by village of birth (*n* = 449) The reference category for mother’s education is ‘less than primary’, and for mother’s age is ‘19–35 years old’

Distance to nearest health facility was associated with three out of four measures of health care utilization (Table 6). One additional kilometer was associated with a 1.2 percentage point (pp) increase in homebirth, a 0.8 pp. decrease in having done at least three antenatal visits, and a 1.2 pp. decrease in skilled assistance at birth. We obtained similar results without control variables (not shown).

**Table 6:** Associations between distance to nearest health facility and maternal health care utilization

Variables	(1) Homebirth	(2) Antenatal	(3) Assistance	(4) Health check
Distance (km)	0.012*** (0.008–0.015)	−0.008** (− 0.014 - -0.002)	− 0.012*** (− 0.016 - -0.008)	− 0.000 (− 0.003–0.003)
First birth	− 0.050** (− 0.093 - -0.007)	− 0.001 (− 0.073–0.072)	0.052** (0.010–0.095)	− 0.014 (− 0.048–0.020)
Twin	−0.083** (−0.149 - -0.017)	− 0.009 (− 0.132–0.114)	0.082** (0.016–0.148)	− 0.042 (−0.096–0.013)
Mother under 19	0.018 (−0.026–0.063)	0.001 (−0.072–0.073)	−0.021 (−0.065–0.023)	0.005 (−0.030–0.039)
Mother over 35	− 0.004 (−0.044–0.036)	0.002 (− 0.049–0.053)	0.003 (−0.036–0.043)	− 0.003 (− 0.032–0.027)
Mother primary ed.	−0.101*** (−0.131 - -0.072)	0.080*** (0.037–0.123)	0.103*** (0.074–0.132)	0.041*** (0.019–0.063)
Mother secondary ed.	−0.334*** (−0.432 - -0.236)	0.173*** (0.045–0.301)	0.335*** (0.237–0.432)	−0.045 (−0.157–0.067)
Constant	0.489*** (0.455–0.523)	0.552*** (0.502–0.602)	0.505*** (0.471–0.539)	0.054*** (0.029–0.079)
Observations	4889	2333	4926	2333
R-squared	0.026	0.011	0.028	0.008

Notes: Coefficients from linear probability models

***p < 0.01

**p < 0.05

*p < 0.1

We found that a 1 km reduction in distance to nearest health facility caused a 2.4% decrease in the probability that a professional checked the mother’s health after a birth (Table 7). We did not find any effect on probability of homebirth, having done at least three antenatal visits, or skilled assistance at birth.

**Table 7:** The effect of changes in distance to nearest health facility on maternal health care utilization

Variables	(1) Homebirth	(2) Antenatal	(3) Assistance	(4) Health check
Distance (km)	0.002 (−0.022–0.025)	0.024 (−0.023–0.071)	−0.005 (−0.027–0.017)	0.024** (0.004–0.044)
First birth	−0.054** (−0.094 - -0.013)	− 0.021 (− 0.100–0.058)	0.057*** (0.016–0.097)	− 0.005 (−0.041–0.031)
Twin	−0.067** (−0.132 - -0.003)	− 0.033 (− 0.166–0.100)	0.065** (0.000–0.130)	− 0.104*** (−0.166 - -0.041)
Mother under 19	0.025 (−0.018–0.068)	0.019 (−0.060–0.098)	−0.027 (−0.070–0.016)	−0.004 (−0.041–0.033)
Mother over 35	−0.016 (−0.055–0.022)	0.017 (−0.038–0.073)	0.011 (−0.028–0.049)	−0.009 (−0.040–0.023)
Mother primary ed.	−0.063*** (−0.094 - -0.032)	0.074*** (0.024–0.124)	0.066*** (0.035–0.096)	0.033** (0.007–0.058)
Mother secondary ed.	−0.223*** (−0.320 - -0.127)	0.166** (0.023–0.308)	0.224*** (0.127–0.320)	−0.052 (−0.175–0.071)
Constant	0.444*** (0.305–0.583)	0.375** (0.086–0.663)	0.583*** (0.448–0.719)	−0.065 (−0.191–0.061)
Children	4889	2333	4926	2333
Villages	449	445	449	409
R-squared	0.280	0.240	0.274	0.247

**p < 0.05

***p < 0.01

Notes: Coefficients from linear probability models with fixed effects for village (n in table) and year (*n* = 4)

## Discussion

### Key results

We found that children born further from health facilities were at higher risk of dying before age five. However, we found no evidence that reductions in distance to the nearest health facility caused by the construction of new health facilities in rural Malawi between 1980 and 2000 caused a change in the risk of children dying before age five. Similarly, we found that pregnant women living further from health facilities were more likely to give birth at home, less likely to have at least three antenatal care visits, and less likely to have skilled assistance at delivery. However, we did not find that reductions in the distance to the nearest health facility changed utilization of any of those three services. We did find, surprisingly, that reductions in distance caused a decrease in the probability that women received a postnatal checkup. Overall, this suggests that the associations between distance and our outcomes are driven by omitted variables, such as local burden of disease or social determinants of health (beyond those controlled for here).

### Limitations

There are several important limitations to this study. First, new health facilities were not constructed in randomly assigned locations. Therefore, it is possible that children born in areas where new facilities were built were systematically more or less likely to die before age five than children in other areas. We used a two-way fixed effects estimation strategy to overcome this endogeneity, but it relied on the assumption that the underlying village-level hazard of under-5 mortality can be modeled as a multiplicative combination of time-invariant village effects and year-specific effects that are common across villages. That assumption implies that, after adjusting for mother’s education, age at birth of her child, whether or not the child is first born, and village characteristics that do not change during the study period, secular trends in under-5 mortality are equivalent across villages.

Second, while we estimated the average treatment effect, new health facilities are likely to produce heterogeneous effects depending on the quality of care provided. The training of the facility staff, the frequency with which they work, and the availability of pharmaceuticals and other medical supplies may vary widely across facilities. We restricted the analysis to dispensaries and maternity/dispensaries, but even within these categories the variability in quality of care may be significant. Variability in quality of care over time – if, for example, new and better health care technologies become available in more recent years – can also lead to heterogeneous effects for similar reasons.

Third, we had limited data on utilization. The MDHS only included those variables for births in the 5 years prior to the survey, so the sample size was roughly one-sixth of that for the mortality analysis. It remains possible that utilization of those services increased in earlier years, or that utilization of other services (e.g., treatment for diarrhea, pneumonia or malaria) increased at any time in the analysis period.

Fourth, the mortality data were retrospective accounts of women who were 15–49 years old in 2000. Children born to women who died were not represented. Because a child is more likely to die if her mother dies, retrospective estimates of under-5 mortality will tend to be underestimates if a substantial proportion of women die between the ages 15 and 49. The potential for bias in this study depends on two factors: the impact of new facilities on adult mortality, and the propensity for new facilities to be built in areas with increasing or decreasing adult mortality. Assume first that new facilities have no impact on adult or child mortality. If new facilities were built in areas where adult mortality is increasing and, thus, under-5 mortality is underestimated, then it will appear that the new facility caused a reduction in under-5 mortality. If, on the other hand, new facilities were built in areas where adult mortality was decreasing, then the opposite will occur. The onset of the HIV epidemic in Malawi in the mid-1980s increased adult mortality substantially [27]. It is unlikely, however, that new facilities were targeted to areas with higher or lower HIV prevalence. The Malawian government in 1980–1998 is unlikely to have possessed adequate capacity to monitor the burden of disease at a high enough spatial and temporal resolution to target new facilities to the areas with the highest burden [13]. Even with sufficient information on disease burden, health systems face a tradeoff between equity and efficiency when deciding where to locate new facilities [28, 29]. Furthermore, there is likely to have been political pressure to use facilities as patronage, targeting areas to gain support rather than reduce disease [30].Another determinant of adult mortality would have been the availability of antiretroviral therapy (ART). In Malawi, ART was not widely available in until after 2004 [31], thus it would not affect the analysis presented here.

Fifth, there is measurement error in our distance measure, which is from the centroid of the village to the health facility. To the extent that households in a village are not located at the centroid, our measure of distance is not accurate. We expect over- and under-estimates from this mismeasurement to be equally likely. Nonetheless, this measurement error would be expected to bias our effect estimates towards the null.

There may also be measurement error from facilities that closed between 1980 and 1998 and were not replaced, because they would not be in the 1998 health facility census. In those cases, we would be overestimating the true distance from the village to that facility. If shorter distances cause lower mortality, then our current analysis will underestimate the effect of reducing distance on mortality. However, we find it unlikely that a large number of facilities were shut down and not replaced during a time period when Malawi’s population nearly doubled, from 6.25 million to 11 million.

### Comparison to similar studies

Previous studies on the relationship between distance to nearest health facility and child mortality show mixed results. A study that combined survey data from 21 countries found that greater distance was associated with higher neonatal mortality, but not mortality at later ages [32]. A study in a rural Kenyan districts with high health facility density found no association between travel time and child mortality [8], nor did a case-control study in rural Gambia [9]. The file drawer problem suggests that studies finding no relationship between distance and mortality are less likely to be published [33].

Studies reporting an association between distance and mortality include a study in rural Burkina Faso which estimated that under-5 mortality was 50% higher at a distance of 4 h walking time to the nearest facility compared with having a facility in the village [4]. A matched pairs study found that the construction of maternity clinics in Indonesia in the mid-1980s reduced infant mortality in the surrounding area by 15% [34]. In South Africa, allowing blacks to utilize facilities that were formerly restricted to whites increased the weight-for-age scores for male infants, but had no effect on female infants [35]. Several other observational studies have found a positive association between distance and under-5 mortality [36–38].

### Generalizability

The external validity of this study benefits from the fact that it used data from villages and health facilities throughout rural Malawi, over a period of 18 years. Nevertheless, this is a study of a single country during a particular phase of history. As discussed above, the effects estimated here likely depend on the quality of health care, the availability of transportation, the demand for health services, and the underlying causes of mortality, among other factors. Data on these variables are scarce in Malawi and other high mortality countries, and more resources should be invested in collecting that information.

## Conclusion

The results presented here suggest that there is more to the story of reducing under-5 mortality than increasing the availability of health infrastructure by reducing distances to the nearest facility. This finding may hold across other low-income, high-mortality countries, particularly in sub-Saharan Africa. More research is needed on the relationship between access to care, quality of care, and perceived quality of care in these settings.

## Supplementary information

**Additional file 1:** Additional results and sensitivity analysis.

## Data Availability

Data and code are available from the corresponding author upon reasonable request.

## Abbreviations

ART: Antiretroviral therapy
MDHS: Malawi Demographic and Health Survey

## References

[1] UNICEF Child mortality data. UNICEF DATA. https://data.unicef.org/topic/child-survival/under-five-mortality/. Accessed 18 Feb 2020.

[2] Sustainable Development Goals. https://sustainabledevelopment.un.org/sdgs. Accessed 18 Feb 2020.

[3] Lohela TJ, Campbell OMR, Gabrysch S (2012). Distance to care, facility delivery and early neonatal mortality in Malawi and Zambia. PLoS One.

[4] Schoeps A, Gabrysch S, Niamba L, Sié A, Becher H (2011). The effect of distance to health-care facilities on childhood mortality in rural Burkina Faso. Am J Epidemiol.

[5] Gabrysch S, Cousens S, Cox J, Campbell OMR. The influence of distance and level of care on delivery place in rural Zambia: a study of linked national data in a geographic information system. PLoS Med. 2011;8(1):e1000394. 10.1371/journal.pmed.1000394. 10.1371/journal.pmed.1000394 PMC3026699 21283606

[6] Nesbitt RC, Lohela TJ, Soremekun S (2016). The influence of distance and quality of care on place of delivery in rural Ghana. Sci Rep.

[7] Hanson C, Gabrysch S, Mbaruku G (2017). Access to maternal health services: geographical inequalities, United Republic of Tanzania. Bull World Health Organ.

[8] Moïsi JC, Gatakaa H, Noor AM (2010). Geographic access to care is not a determinant of child mortality in a rural Kenyan setting with high health facility density. BMC Public Health.

[9] Rutherford ME, Dockerty JD, Jasseh M (2009). Access to health care and mortality of children under 5 years of age in the Gambia: a case-control study. Bull World Health Organ.

[10] Kim ET, Singh K, Speizer IS, Angeles G, Weiss W (2019). Availability of health facilities and utilization of maternal and newborn postnatal care in rural Malawi. BMC Pregnancy Childbirth.

[11] Rutherford ME, Mulholland K, Hill PC (2010). How access to health care relates to under-five mortality in sub-Saharan Africa: systematic review. Tropical Med Int Health.

[12] World Bank. World Bank data catalog. 2020 https://data.worldbank.org/indicator. Accessed 18 Apr 2019.

[13] Banda EEN, Simukonda HP (1994). The public/private mix in the health care system in Malawi. Health Policy Plan.

[14] Cortez R, Saadat S, Chowdhury S, Sarker I. Maternal and Child Survival: Findings from five countries experience in addressing maternal and child health challenges. Washington, D.C.: The World Bank; 2014. https://ideas.repec.org/p/wbk/hnpdps/91294.html. Accessed 18 Feb 2020.

[15] National Safe Motherhood Task Force of Malawi. Making motherhood safe for Malawian women. Ministry of Health, Llongwe, Malawi. 1995.

[16] Kulmala T, Vaahtera M, Rannikko J (2000). The relationship between antenatal risk characteristics, place of delivery and adverse delivery outcome in rural Malawi. Acta Obstet Gynecol Scand Orig Artic.

[17] Walsh A, Matthews A, Manda-Taylor L (2018). The role of the traditional leader in implementing maternal, newborn and child health policy in Malawi. Health Policy Plan.

[18] Doherty T, Zembe W, Ngandu N, et al. Assessment of Malawi’s success in child mortality reduction through the lens of the catalytic initiative integrated health systems strengthening programme: retrospective evaluation. J Glob Health. 2015;5. 10.7189/jogh.05.020412 PMC4652924 26649176

[19] Jahn A, Floyd S, Crampin AC (2010). Declining child mortality in northern Malawi despite high rates of infection with HIV. Bull World Health Organ.

[20] National Statistical Office, ORC Macro. Malawi Demographic and Health Survey 2000. Zomba; 2001.

[21] Cox DR (1972). Regression models and life-tables. J R Stat Soc Ser B Methodol.

[22] Therneau TM, Grambsch PM. The Cox model. In: Modeling survival data: extending the Cox model. New York: Springer; 2000. p. 39–77.

[23] Allison PD. Fixed effects regression models. Thousand Oaks: SAGE publications; 2009.

[24] Grambsch PM, Therneau TM (1994). Proportional hazards tests and diagnostics based on weighted residuals. Biometrika.

[25] Box-Steffensmeier JM, Jones BS. Event History Modeling: A Guide for Social Scientists. Cambridge: Cambridge University Press; 2004.

[26] Benjamini Y, Hochberg Y. Controlling the false discovery rate: a practical and powerful approach to multiple testing. J R Stat Soc Ser B Methodol. 1995;57(1):289–300.

[27] UNAIDS (2010). Report on the Global AIDS Epidemic.

[28] Abiiro GA, Mbera GB, De Allegri M (2014). Gaps in universal health coverage in Malawi: a qualitative study in rural communities. BMC Health Serv Res.

[29] Norheim OF (2015). Ethical perspective: five unacceptable trade-offs on the path to universal health coverage. Int J Health Policy Manag.

[30] Wild L, Cammack D. The supply and distribution of essential medicines in Malawi. Overseas Dev Inst Work Pap. 2013. https://www.odi.org/sites/odi.org.uk/files/odi-assets/publications-opinion-files/8176.pdf.

[31] WHO, UNICEF, UNAIDS. Global update on HIV treatment 2013: results, impact and opportunities. Geneva: World Health Organization; 2013. http://www.who.int/hiv/pub/progressreports/update2013/en/index.html. Accessed 30 Sept 2013.

[32] Karra M, Fink G, Canning D (2017). Facility distance and child mortality: a multi-country study of health facility access, service utilization, and child health outcomes. Int J Epidemiol.

[33] Rosenthal R (1979). The file drawer problem and tolerance for null results. Psychol Bull.

[34] Frankenberg E (1995). The effects of access to health care on infant mortality in Indonesia. Health Transit Rev.

[35] Tanaka S (2014). Does abolishing user fees lead to improved health status? Evidence from post-apartheid South Africa. Am Econ J Econ Pol.

[36] Schellenberg JRA, Mrisho M, Manzi F (2008). Health and survival of young children in southern Tanzania. BMC Public Health.

[37] Van den Broeck J, Eeckels R, Massa G (1996). Maternal determinants of child survival in a rural African community. Int J Epidemiol.

[38] Magnani RJ, Rice JC, Mock NB, Abdoh AA, Mercer DM, Tankari K (1996). The impact of primary health care services on under-five mortality in rural Niger. Int J Epidemiol.

