# Supplementary material for: The effects of changes in distance to nearest health facility on under-5 mortality and health care utilization in rural Malawi, 1980–1998
Source: BMC Health Serv Res. 2020 Sep 24;20:899. doi: 10.1186/s12913-020-05738-w (PMC7517642; doi:10.1186/s12913-020-05738-w)
Supplement: Supplementary file 1 — Additional file 1: Additional results and sensitivity analysis. [file 12913_2020_5738_MOESM1_ESM.docx]

**Additional File 1**

**The effects of changes in distance to nearest health facility on under-5 mortality and health care utilization in rural Malawi, 1980-1998**

**Authors:** John Quattrochi^a*^, Kenneth Hill^c^, Joshua A Salomon^b^, Marcia C. Castro^d^

**Affiliations:**

^a^Simmons University, Department of Public Health, 300 The Fenway, Boston, MA, 02115, USA
[john.quattrochi@simmons.edu](mailto:john.quattrochi@simmons.edu)
^b^Harvard T.H. Chan School of Public Health, Department of Global Health and Population, 655 Huntington Ave., Boston, MA, 02115, USA
kenneth_hill_1@yahoo.com
^c^Stanford University, Center for Health Policy and Center for Primary Care Outcomes and Research, 616 Serra Street, Stanford, CA 94305, USA
salomon1@stanford.edu
^d^Harvard T.H. Chan School of Public Health, Department of Global Health and Population, 655 Huntington Ave., Boston, MA, 02115, USA
mcastro@hsph.harvard.edu

*Corresponding author: [john.quattrochi@simmons.edu](mailto:john.quattrochi@simmons.edu)

# Test for recall bias

Since the outcome was measured via retrospective reporting of events, we checked the data for indications of recall bias. Selective omission of births that did not survive, for example, would lead to underestimation of mortality rates. If early neonatal deaths (deaths in the first week of life) were selectively underreported, the result would be an unusually low ratio of deaths under seven days to all neonatal deaths (deaths in the first 28 days of life) and an unusually low ratio of neonatal to infant deaths (deaths in the first year of life). Births further back in time may be more likely to be omitted; thus a common data quality check is to examine the ratios over time. The MDHS report shows that the proportion of neonatal deaths in the first week of life was high (67%) and was roughly constant over the 20 years before the survey (66-71%). In addition, the proportion of infant deaths during the first month of life (42%) was comparable to that observed in other similar countries 15 and was stable over the 20 years before the survey (38-44%). Therefore we concluded that recall bias is not a significant concern in this study.

# Additional results

Child survival by age followed a typical pattern for high mortality areas (Figure A1). Five percent of children died in the first month, followed by a flattening out of the hazard rate over the next 59 months of life. Over 20% of children on average over the study period died before age 5.

**Figure A1 Kaplan-Meier curve for analysis sample** time unit = month

The number of deaths per year (Figure A2) reflects both the change in number of children born per year and the change in the death rate over time. Deaths increased steadily from a low of 46 in 1980 to 275 in 1994, then decreased for two years before increasing again to the maximum of 312 in 1999.

**Figure A2. Deaths per year in analysis sample**


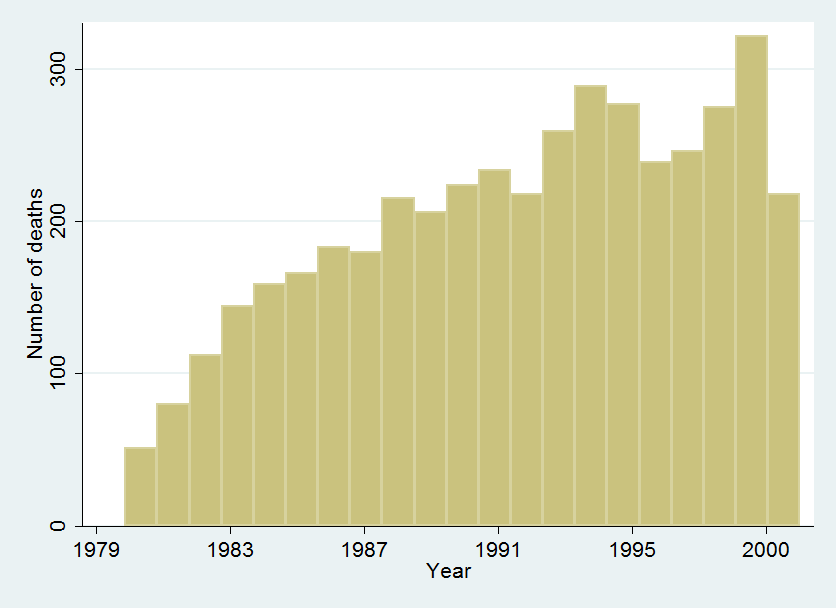


# Tests of the proportional hazards assumption

We applied the Grambsch and Therneau test of the proportional hazards assumption to our preferred causal model of the effect of reductions in distance to nearest health facility on under-5 mortality. It is a Cox proportional hazards model with categorical distance and full controls. The test identified one control variable as violating the proportional hazards assumption, namely, that the child was a twin (Table A1). However, visual inspection of the plots of the scaled Schoenfeld residuals for that variable against survival time revealed that the change in slope was not substantivey meaninful (Figure A3). Furthermore, sensitivity analysis shows that models without the twin covariate produce similar results (Table A2). We concluded that the proportional hazards assumption is reasonable for this analysis.

**Table A1. Grambsch and Therneau test for non-proportional hazards applied to full model of under-5 mortality**

| Variable | rho | chi2 | df | Prob>chi2 |
| --- | --- | --- | --- | --- |
| >10km to 5-10km | 0.00588 | 0.13 | 1 | 0.7182 |
| >10km to 2-5km | -0.01206 | 0.54 | 1 | 0.462 |
| >10km to <2km | 0.01153 | 0.59 | 1 | 0.4431 |
| 5-10km to 2-5km | 0.02355 | 2.35 | 1 | 0.1255 |
| 5-10km to <2km | -0.01593 | 0.99 | 1 | 0.3187 |
| 2-5km to <2km | -0.00115 | 0.01 | 1 | 0.942 |
| 1981 | 0.00069 | 0 | 1 | 0.9657 |
| 1982 | 0.00271 | 0.03 | 1 | 0.8663 |
| 1983 | 0.00194 | 0.01 | 1 | 0.9033 |
| 1984 | 0.01141 | 0.5 | 1 | 0.4791 |
| 1985 | 0.00501 | 0.1 | 1 | 0.7557 |
| 1986 | 0.00876 | 0.3 | 1 | 0.5857 |
| 1987 | 0.00674 | 0.18 | 1 | 0.6751 |
| 1988 | 0.01155 | 0.51 | 1 | 0.4735 |
| 1989 | 0.01608 | 0.99 | 1 | 0.3197 |
| 1990 | -0.00468 | 0.08 | 1 | 0.7713 |
| 1991 | -0.00302 | 0.03 | 1 | 0.8521 |
| 1992 | -0.00454 | 0.08 | 1 | 0.7786 |
| 1993 | -0.00511 | 0.1 | 1 | 0.7522 |
| 1994 | -0.01134 | 0.49 | 1 | 0.4821 |
| 1995 | -0.00282 | 0.03 | 1 | 0.8617 |
| 1996 | -0.00233 | 0.02 | 1 | 0.8854 |
| 1997 | -0.00452 | 0.08 | 1 | 0.7797 |
| 1998 | -0.01544 | 0.91 | 1 | 0.3398 |
| February | 0.00092 | 0 | 1 | 0.9542 |
| March | 0.00172 | 0.01 | 1 | 0.9142 |
| April | 0.00494 | 0.1 | 1 | 0.7577 |
| May | 0.00517 | 0.1 | 1 | 0.7473 |
| June | 0.00614 | 0.15 | 1 | 0.7009 |
| July | -0.00031 | 0 | 1 | 0.9846 |
| August | 0.02877 | 3.22 | 1 | 0.0726 |
| September | 0.01238 | 0.6 | 1 | 0.4391 |
| October | 0.0103 | 0.42 | 1 | 0.5185 |
| November | 0.01419 | 0.79 | 1 | 0.3739 |
| December | 0.00511 | 0.1 | 1 | 0.7492 |
| First birth | -0.02108 | 1.87 | 1 | 0.1718 |
| Twin | -0.09202 | 32.75 | 1 | 0 |
| Mother under 19 | -0.02899 | 3.46 | 1 | 0.0628 |
| Mother over 35 | -0.00862 | 0.28 | 1 | 0.5969 |
| Mother primary ed. | 0.00816 | 0.27 | 1 | 0.6066 |
| Mother secondary ed. | -0.0093 | 0.33 | 1 | 0.5657 |
| Global test |  | 74.54 | 41 | 0.0011 |

**Figure A3. Scaled Schoenfeld residuals for ‘twin’ vs survival time**

**Table A2. Sensitivity analysis for the effect of changes in distance to nearest health facility on under-5 mortality, categorical distance**

|  | (1) |  | (2) |  |
| --- | --- | --- | --- | --- |
| VARIABLES | U5M | adj. p-value | U5M | adj. p-value |
|  |  |  |  |  |
| >10km to 5-10km | 1.084 |  | 1.092 |  |
|  | (0.837 - 1.405) | 0.70 | (0.844 - 1.413) | 0.75 |
| >10km to 2-5km | 1.354 |  | 1.297 |  |
|  | (0.914 - 2.007) | 0.39 | (0.879 - 1.914) | 0.57 |
| >10km to <2km | 0.922 |  | 1.020 |  |
|  | (0.461 - 1.846) | 0.82 | (0.509 - 2.045) | 0.96 |
| 5-10km to 2-5km | 0.789 |  | 0.807 |  |
|  | (0.604 - 1.029) | 0.39 | (0.620 - 1.052) | 0.57 |
| 5-10km to <2km | 0.891 |  | 0.910 |  |
|  | (0.588 - 1.351) | 0.70 | (0.600 - 1.379) | 0.79 |
| 2-5km to <2km | 1.336 |  | 1.304 |  |
|  | (0.660 - 2.705) | 0.70 | (0.642 - 2.648) | 0.75 |
| First birth | 1.011 |  | 0.964 |  |
|  | (0.918 - 1.114) |  | (0.875 - 1.061) |  |
| Twin | 3.050*** |  |  |  |
|  | (2.691 - 3.457) |  |  |  |
| Mother under 19 | 1.389*** |  | 1.393*** |  |
|  | (1.264 - 1.527) |  | (1.268 - 1.531) |  |
| Mother over 35 | 0.995 |  | 1.015 |  |
|  | (0.889 - 1.114) |  | (0.908 - 1.136) |  |
| Mother primary ed. | 0.865*** |  | 0.869*** |  |
|  | (0.804 - 0.931) |  | (0.807 - 0.935) |  |
| Mother secondary ed. | 0.452*** |  | 0.460*** |  |
|  | (0.283 - 0.720) |  | (0.289 - 0.733) |  |
| Year dummies? | YES |  | YES |  |
| Month dummies? | YES |  | YES |  |
|  |  |  |  |  |
| Total children | 18,714 |  | 18,714 |  |
| Total deaths | 3,887 |  | 3,887 |  |
| Total child-months | 737,547 |  | 737,547 |  |

Notes: Hazard ratios from Cox proportional hazards models, with baseline hazard stratified by village of birth (n=449) The reference category for mother’s education is ‘less than primary’, and for mother’s age is ’19-35 years old.’

# Sensitivity analysis with logarithmic distance

As an additional sensitivity analysis, we tested the association between the logarithm of distance and child mortality (Table A3). A ten-fold increase in distance was associated with a 5.7% increase in hazard of death (95%CI 1.3% to 10.3%). Adding controls for year and month reduced the hazard ratio, and the association was no longer significant at p<0.05. These results are similar to the results with linear distance.

**Table A2. Sensitivity analysis for the association between distance to nearest health facility on under-5 mortality, logarithmic distance**

|  |  |  |  |
| --- | --- | --- | --- |
|  | (1) | (2) | (3) |
| Variables | U5M | U5M | U5M |
|  |  |  |  |
| log(Distance) | 1.057*** | 1.034 | 1.035 |
|  | (1.013 - 1.103) | (0.991 - 1.079) | (0.992 - 1.079) |
|  |  |  |  |
| Year dummies? | NO | YES | YES |
| Month dummies? | NO | NO | YES |
|  |  |  |  |
| Total children | 18,714 | 18,714 | 18,714 |
| Total deaths | 3,887 | 3,887 | 3,887 |
| Total child-months | 737,547 | 737,547 | 737,547 |

Notes: U5M = under-5 mortality. Hazard ratios (95% confidence intervals) from proportional hazards models. Distance is logarithmic distance to nearest health facility from village centroid. The coefficient on the distance variable represents the HR for a one-kilometer increase in distance. The reference category for mother’s education is ‘less than primary’, and for mother’s age is ’19-35 years old.’ *** p<0.01, ** p<0.05, * p<0.1
